# Presence of caffeine reversibly interferes with efficacy of acupuncture-induced analgesia

**DOI:** 10.1038/s41598-017-03542-x

**Published:** 2017-06-13

**Authors:** Takumi Fujita, Changyong Feng, Takahiro Takano

**Affiliations:** 1 Eastman Institute for Oral Health, University of Rochester, Rochester, NY USA; 2 Department of Biostatistics and Computational Biology, University of Rochester, Rochester, NY USA

## Abstract

Acupuncture is an alternative treatment for wide spectrum chronic pain. However, its validity remains controversial due to the disputed efficacy assessed in various clinical studies. Moreover, variability amongst individuals complicates the predictability of outcome, which impedes the integration of acupuncture into mainstream pain management programs. In light of our previous finding that the analgesic effect of acupuncture is mediated by adenosine A1 receptor activation at the acupuncture point, we here report that in acute and chronic animal pain models, oral intake of caffeine, a potent adenosine receptor antagonist, interferes with acupuncture analgesia, even at a low dose. Local administration of caffeine at the acupuncture point was sufficient to eliminate the analgesic effect, dismissing the systemic action of caffeine. Such interference was reversible, as caffeine withdrawal fully restored the efficacy of acupuncture by the next day, and long-term exposure to caffeine did not alter A1 receptor expression at the acupuncture point. Combined, these data indicate that a trace amount of caffeine can reversibly block the analgesic effects of acupuncture, and controlling caffeine consumption during acupuncture may improve pain management outcomes.

## Introduction

Analgesia is one of the most widely accepted effects of acupuncture. However, acupuncture’s efficacy beyond the placebo effect still remains controversial, primarily because of an incomplete understanding of its biological basis. The credibility is further diminished by the high variability of the efficacy, ranging from complete pain relief to no effect at all. Thus, acupuncture is often used as a last treatment option, even though pain reduction as a result of acupuncture has been demonstrated in many human and veterinary patients, without adverse side effects that are often present with medications.

﻿We previously showed that, in an animal model, the analgesic effect of acupuncture is mediated by a steep increase in extracellular adenosine levels at the acupuncture point and subsequent local activation of the adenosine A1 receptor^1^. The A1 receptors responsible for mediating analgesia are localized in the vicinity of the acupuncture point rather than at distant central nervous system. This is evident since local administration of the A1 receptor agonist, 2-Chloro-N6-cyclopentyladenosine (CCPA), at the acupuncture point also resulted in analgesia, while acupuncture applied to the contralateral leg to the site of pain failed to reduce pain^1^. Such a transient adenosine increase was also present with acupuncture in humans^2^.

Adenosine receptor activation during acupuncture analgesia is potentially impacted by caffeine, which is a potent adenosine receptor antagonist. Caffeine is present in a wide variety of foods and drinks, and it is estimated that around 90% of the US population consumes caffeine daily^3^. Even though the average half-life of caffeine in plasma is 4–5 hours, a detectable amount of caffeine stays in the body for more than 12 hours after drinking a cup of coffee^4–6^. Therefore, it is likely that a residual amount of caffeine could be present at the time of acupuncture treatment in those who ingest caffeine daily.

In our animal model, genetic removal of the adenosine A1 receptor completely abolished the analgesic effect of acupuncture^1^. While the inhibitory constant (Ki) of caffeine at the A1 receptor is reported to be 10–44 µM^7^, a cup of coffee would result in a peak caffeine concentration of ~10 µM in the plasma^8^. Thus, a low level of residual caffeine could still potentially interfere with the mechanism of adenosine receptor-mediated analgesia during acupuncture. Therefore, we examined whether caffeine consumption reduces the efficacy of acupuncture analgesia in both acute and chronic pain models. We also tested whether such inhibition could be reversed if caffeine was withdrawn.

## Results

### Caffeine interferes with the analgesic effect of acupuncture in acute inflammatory pain

**Figure 1:**
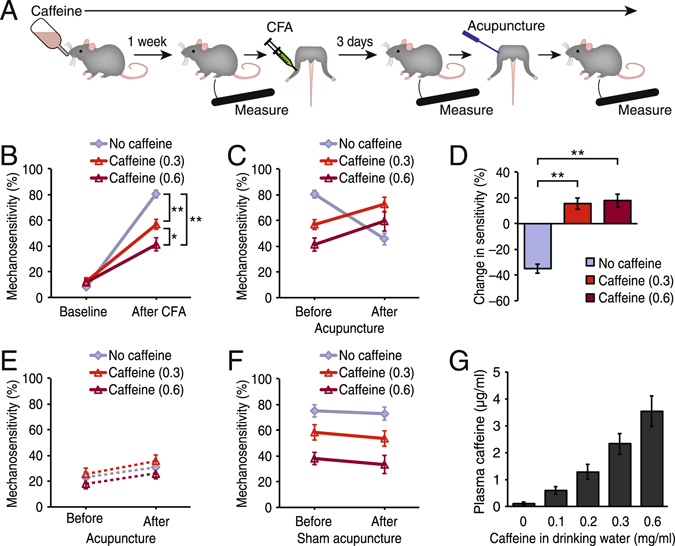
Caffeine interferes with the effect of acupuncture on acute inflammatory pain. (**A**) Schematic diagram outlining the experimental process of caffeine oral administration and behavioral measurements in the acute inflammatory pain model. Mice were acclimated to drinking water containing caffeine for 1 week. Baseline mechanical sensitivity was measured using von Frey filament, then pain was induced by administration of complete Freund’s adjuvant (CFA) in the ankle joint. Three days later, mechanical hypersensitivity was measured before an acupuncture treatment was given to the affected hind leg, followed by another measurement to obtain the changes in mechanical sensitivity by acupuncture treatment with or without caffeine administration. (**B**) Pain induction by CFA under caffeine consumption. Mice receiving normal drinking water (No caffeine, blue grey) showed prominent mechanosensitivity 3 days after CFA administration. Mice treated with the drinking water containing caffeine at the doses of 0.3 mg/ml (red) and 0.6 mg/ml (dark red) also exhibited pain behavior, but with lower intensity compared to the no caffeine group. N = 15–16; *p < 0.05, **p < 0.01. (**C**) Mechanical sensitivity before and after acupuncture treatment. Mice with normal drinking water showed a reduction of sensitivity after acupuncture treatment. However, the same acupuncture treatment failed to reduce pain in mice receiving the drinking water supplemented with caffeine (0.3 and 0.6 mg/ml). N = 15–16. (**D**) Summary histogram comparing the changes in mechanical sensitivity by acupuncture amongst three groups that are shown in panel C. In the no caffeine group, acupuncture reduced pain, but the effect was reversed when the mice were administered caffeine. **p < 0.01. (**E**) Mechanical sensitivity in the contralateral leg to the inflammation. In the contralateral leg, no pain was induced by CFA. Acupuncture to the ipsilateral leg did not significantly alter the mechanical sensitivity of the contralateral leg (p = 0.94). (**F**) Mechanical sensitivity before and after sham acupuncture treatment. All three groups (No caffeine, 0.3 and 0.6 mg/ml of caffeine groups) showed no reductions in sensitivity following sham acupuncture treatment. N = 14–16. (**G**) Caffeine in mouse plasma. The average caffeine concentration in the plasma increased corresponding to the caffeine concentration in the drinking water. N = 16–21. Displayed are means ± S.E.M. in **B**–**G**.

Acute inflammatory pain in the left hind limb ankle joints was induced by intra-articular administration of complete Freund’s adjuvant (CFA). Mechanical sensitivity was measured using the von Frey filament touch test 3 days after CFA injection (Fig. 1A). Mice that received drinking water without caffeine exhibited an increase in mechanosensitivity, defined as the percent of positive responses to the filament touch in a set of trials, from 8.1 ± 1.1% to 80.6 ± 3.1% in the ipsilateral leg to the inflammation (Fig. 1B). Similarly, increased mechanosensitivity was detected in a group of mice received drinking water containing 0.3 mg/ml of caffeine, which is comparable to 4–5 cups of coffee in a man per day^9^, as well as in another group that received 0.6 mg/ml of caffeine. Although all groups developed hyper-mechanosensitivity in the ipsilateral leg, the degree of response was dose-dependently decreased in the caffeine groups compared to the no caffeine group (p < 0.0001) (Fig. 1B). Following the mechanosensitivity measurement, animals received acupuncture treatment, in which an acupuncture needle was applied to ST36 acupuncture point of the ipsilateral leg for 20 min with gentle manipulation, and further measurements were taken to evaluate the effects of the treatment. The no caffeine group showed a 35.0 ± 3.5% reduction in sensitivity following acupuncture treatment (p < 0.0001 compared to Before acupuncture) (Fig. 1C,D). In contrast, the analgesic effect of acupuncture was not observed in either of the caffeine-drinking groups. Rather, acupuncture treatment increased the mechanosensitivity by 15.6 ± 4.5% in the 0.3 mg/ml caffeine group and 18.0 ± 5.0% in the 0.6 mg/ml caffeine group (p < 0.003 compared to Before acupuncture) (Fig. 1C,D). The responses to the filament touch in the contralateral limb were unaffected by caffeine (p = 0.13 compared to Before acupuncture; p = 0.94 among the three groups), suggesting that caffeine does not alter motor functions (Fig. 1E). The increase or decrease of mechanosensitivity was not due to the needle insertion or the stress of prolonged restraints during treatment, as sham acupuncture, in which the same acupuncture needle was inserted for 20 min without manual stimulation, did not alter the mechanosensitivity in all groups (−2.1 ± 4.5%, −4.8 ± 8.5% and −4.8 ± 5.9% change in No caffeine, 0.3 mg/ml caffeine and 0.6 mg/ml caffeine groups, respectively; p > 0.4 compared to Before sham acupuncture) (Fig. 1F). The amount of caffeine present in the plasma at the time of the acupuncture session was on average 2.3 ± 0.4 µg/ml in mice that received 0.3 mg/ml caffiene, and 3.5 ± 0.6 µg/ml in those that received 0.6 mg/ml caffeine (Fig. 1G).

### Caffeine interferes with the analgesic effect of acupuncture in chronic pain in a dose dependent manner

**Figure 2:**
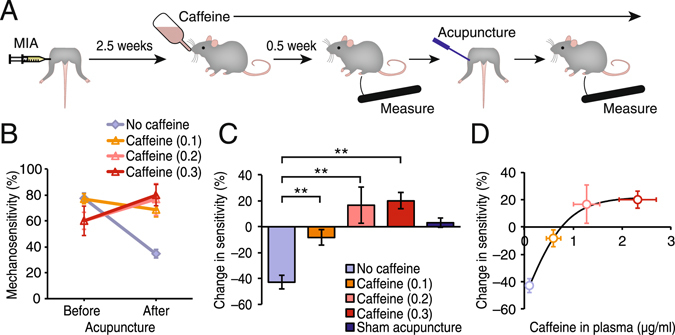
Caffeine interferes with the effect of acupuncture on chronic osteoarthritis pain. (**A**) Schematic diagram illustrating the major steps of the experimental procedure in the chronic joint pain model. Pain was induced by monosodium iodoacetate (MIA) in a knee joint 3 weeks prior to the measurement of mechanical sensitivity with von Frey filament. After pain induction (2.5 weeks), mice were acclimated to drinking water containing caffeine for 3–4 days. Mechanical sensitivity was measured twice, before and after acupuncture treatment given to the affected hind leg in awake mice. (**B**) Comparison of mechanical sensitivity before and after acupuncture treatment. Mice that received normal drinking water (No caffeine, blue grey) showed significant decrease in sensitivity following acupuncture treatment, whereas such reduction in sensitivity was absent in mice drinking caffeine-containing water (0.1 mg/ml, orange; 0.2 mg/ml, pink; 0.3 mg/ml, red). Sham acupuncture treatment did not alter the mechanical sensitivity (dark blue). N = 5–14. (**C**) Summary histogram of changes in mechanical sensitivity by acupuncture that are shown in panel B. Similar to the acute pain model, acupuncture decreased the mechanical sensitivity of mice in the no caffeine group in the chronic osteoarthritis pain model. The anti-nociceptive effect of acupuncture was reversed when the mice were administered caffeine. Sham acupuncture did not alter the mechanosensitivity. N = 5–16. **p < 0.01. **(D)** Dose-response histogram correlating plasma levels of caffeine and acupuncture-induced changes in mechanosensitivity. Displayed are means ± S.E.M. in **B**–**D**.

As shown in Fig. 1, inhibition of acupuncture analgesia by caffeine was robust in the acute pain model. We therefore next tested the effect of lower caffeine doses in the chronic pain model. Knee osteoarthritis was induced by administering monosodium iodoacetate (MIA) in the hind limb knee joints of mice, in which chronic pain developed in 2.5 weeks. Mice were then given drinking water with either no or low caffeine (0.1, 0.2, or 0.3 mg/ml) (Fig. 2A). At 3 weeks after pain induction, all four groups showed an increase in mechanical sensitivity (Fig. 2B). Similar to the CFA-induced ankle joint pain, MIA-induced knee arthritis pain was attenuated by acupuncture treatment (42.9 ± 5.2% reduction, p < 0.0001 compared to Before acupuncture) (Fig. 2B,C). A low dose of 0.1 mg/ml caffeine in the drinking water resulted in a small but detectable caffeine level in the plasma (0.6 ± 0.1 µg/ml) (Fig. 1F). Even at this low concentration, the analgesic effect of acupuncture was blocked (−8.3 ± 6.1% change, p = 0.19 compared to Before acupuncture, p < 0.001 compared to After acupuncture in No caffeine group) (Fig. 2B,C). In the 0.2 and 0.3 mg/ml caffeine groups, acupuncture appeared to worsen the pain, with mechanosensitivity enhanced by 16.7 ± 13.9% (p = 0.30 compared to Before acupuncture) and 20.0 ± 6.2% (p = 0.03 compared to Before acupuncture), respectively (Fig. 2B,C). As in the acute pain model, sham acupuncture treatment did not alter the mechanosensitivity (3.1 ± 3.8% change, p = 0.42 compared to Before sham acupuncture) (Fig. 2C). Changes in mechanosensitivity after acupuncture plotted against the plasma caffeine levels demonstrate a dose-dependent effect, with an inhibitory concentration (IC50) calculated as 0.51 µg/ml (R^2^ = 0.985) (Fig. 2D).

### The presence of caffeine at the acupuncture site abolishes the analgesic effect of acupuncture

**Figure 3:**
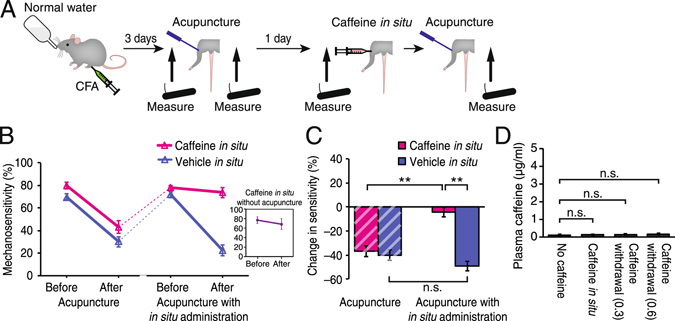
The presence of caffeine at the acupuncture point at the time of acupuncture treatment interferes with the analgesic effects of acupuncture. (**A**) Schematic diagram outlining the two consecutive day behavioral examination and the timing of local caffeine administration. Pain was induced by CFA administration in the ankle joint 3 days prior to the measurements of mechanical sensitivity, before and after acupuncture. On the following day, another set of pain measurements was taken in conjunction with local administration of caffeine (100 µM, 5 µl) at the acupuncture point. (**B**) Mechanical sensitivity repeatedly measured on two consecutive days with or without caffeine administration at acupuncture point. Acupuncture reduced mechanical sensitivity in mice without caffeine (Before and After Acupuncture, both magenta and blue lines). Conversely, mechanical sensitivity remained high after acupuncture when caffeine was directly administered at the acupuncture point prior to acupuncture (Caffeine *in situ* in Acupuncture with *in situ* administration group, magenta). N = 16. Vehicle administration did not alter the acupuncture-induced analgesia (Vehicle *in situ* in Acupuncture with *in situ* administration group, blue). N = 16. ***Insert***: Caffeine *in situ* administration without acupuncture did not alter mechanosensitivity. N = 8. (**C**) Summary of acupuncture-induced changes in mechanosensitivity shown in panel B. Local administration of caffeine abolished the analgesic effect of acupuncture. **p < 0.001; n.s., p > 0.1. (**D**) Caffeine in mice plasma under the conditions of no caffeine, 15 min after caffeine administeration at acupuncture point (Caffeine *in situ*), or 24 hours after caffeine withdrawal from drinking water (0.3 or 0.6 mg/ml). N = 15–20; n.s., p > 0.1. Displayed are means ± S.E.M. in **B**–**D**.

A previous report demonstrated that the adenosine A1 receptor responsible for acupuncture-induced anti-nociception is located at the acupuncture point^1^. However, orally consumed caffeine also acts on adenosine receptors present in other tissues, including central nervous and cardiovascular systems. Therefore, it is unclear whether caffeine blocks activation of A1 receptors in the vicinity of the acupuncture point, or if there is a certain systemic effect of caffeine that suppresses acupuncture analgesia. To address this question, we next administered caffeine at the acupuncture point instead of oral administration (Fig. 3A). On the first day of pain measurement, mice in the no caffeine condition were given acupuncture treatment to confirm the presence of the analgesic effect (−36.9 ± 4.4% change by acupuncture) (Fig. 3A, B). As previously reported, acupuncture analgesia was temporal^1^, and by the next day the mechanosensitivity returned to pre-acupuncture levels (80.1 ± 3.0% to 78.1 ± 2.1%) (Fig. 3B). On the second day of pain assessment, a small volume of high concentration caffeine (100 µM; 5 µl) was injected to the acupuncture point prior to the acupuncture session. The administered concentration was high to ensure complete local blockage of adenosine receptors, without causing neurotoxicity^10^. The presence of caffeine at the acupuncture point abolished the analgesic effect (−4.4 ± 4.1% change, p = 0.30 compared to Before acupuncture, p < 0.0001 compared to After acupuncture on the day before) (Fig. 3B,C). In contrast, prior administration of vehicle instead of caffeine did not disrupt the acupuncture analgesia (−49.4 ± 3.9% change, p < 0.001 compared to Before acupuncture) (Fig. 3B,C). *In situ* administration of caffeine alone without subsequent manual acupuncture did not alter the mechanosensitivity of the animals (−8.3 ± 6.3% change, p = 0.23 compared to Before caffeine administration) (Fig. 3B *insert*). The effect of local administration of caffeine was not systemic, as caffeine was not detected in the plasma of these animals (p = 0.14 compared to No caffeine group) (Fig. 3D). Therefore, the local presence of caffeine was sufficient to eliminate the efficacy of acupuncture, dismissing the involvement of caffeine’s action in the central nervous and cardiovascular systems.

### Caffeine habituation does not alter the mechanism of acupuncture-induced analgesia

**Figure 4:**
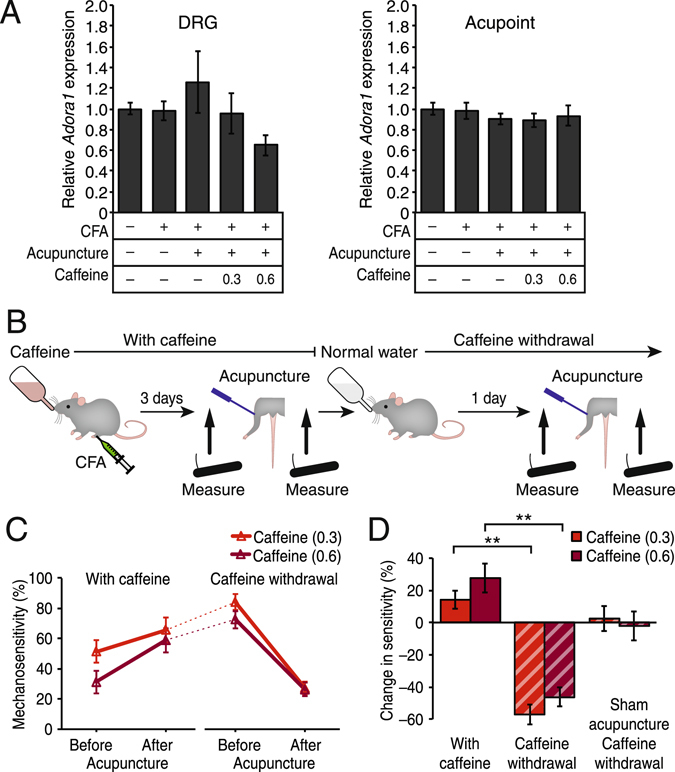
Caffeine did not modulate expression of the adenosine A1 receptor in DRG and tissue around the acupuncture point. (**A**) qPCR analysis of *Adora1* expression relative to untreated control mice (without CFA administration, caffeine drinking, and acupuncture treatment) in the ipsilateral L3-L5 dorsal root ganglia (DRG, *left panel*) and muscle tissue near the acupuncture point (Acupoint, *right panel*). Expression of *Adora1* was not significantly altered by acupuncture treatment and chronic oral administration of caffeine. N = 5–8; p > 0.1. (**B**) Schematic diagram depicting how the effect of caffeine withdrawal was evaluated. Mice were acclimated to drinking water containing caffeine for 1 week. Pain was induced by CFA administered in the ankle joint 3 days prior to the measurements of mechanical sensitivity, before and after acupuncture. Following the measurement of after-acupuncture condition, caffeine-containing water was replaced with normal water for 24 hours (Caffeine withdrawal), and then sensitivity was re-assessed before and after acupuncture. (**C**) Mechanical sensitivity repeatedly measured on two consecutive days with or without systemic caffeine. Withdrawal of caffeine (0.3 mg/ml, red; 0.6 mg/ml, dark red) restored the efficacy of acupuncture. N = 7–8. (**D**) Summary histogram of acupuncture-induced changes in sensitivity that are shown in panel F. The analgesic effect of acupuncture was blocked when the mice were administered caffeine via drinking water (With caffeine) but was fully restored after caffeine withdrawal (Caffeine withdrawal, bars with hatched lines). Sham acupuncture after withdrawal did not affect the mechanosensitivity. N = 7–8. **p < 0.001. Displayed are means ± S.E.M. in **A**,**C**,**D**.

Persistent inhibition of A1 receptors by long-term consumption of caffeine might decrease A1 receptor expression at the acupuncture point, which may influence the efficacy of acupuncture. Therefore, we next measured the expression of the A1 receptor gene, *Adora1*, both in the muscle tissue around the acupuncture point (acupoint) and in the sciatic nerve dorsal root ganglia (DRG), which send axons underneath the acupoint. Induction of pain by CFA administration as well as acupuncture treatment did not alter *Adora1* expression at either site (Fig. 4A). In mice drinking 0.3 mg/ml of caffeine, *Adora1* expression was also unaffected (Fig. 4A). At the high dose of caffeine (0.6 mg/ml), a slight non-significant decrease in *Adora1* expression was detected in the DRG (p = 0.21), but there was no change at the acupoint (p = 0.75) (Fig. 4A). Thus, inhibition of acupuncture analgesia by caffeine was unlikely due to decreased A1 receptor expression at the DRG or the acupoint.

Next we tested whether the analgesic effects of acupuncture could be restored following caffeine removal. Caffeine is known to induce mild physical dependency associated with sustained physiological and molecular changes after daily consumption^11–13^. Thus, certain long-term changes caused by caffeine habituation may influence acupuncture treatment. First, mechanosensitivity in mice in the caffeine conditions (0.3 and 0.6 mg/ml) were assessed before and after acupuncture. We then replaced the drinking water containing caffeine with normal water and re-evaluated the sensitivity in the same mice on the second day following caffeine withdrawal (Fig. 4B). After 24 hours, there were no detectable levels of caffeine present in the plasma (p = 0.37 for 0.3 mg/ml and p = 0.34 for 0.6 mg/ml caffeine groups compared to No caffeine group) (Fig. 3D). Caffeine withdrawal increased mechanosensitivity before acupuncture treatment (51.4 ± 7.4% to 84.3 ± 5.3% and 31.3 ± 7.7% to 72.5 ± 5.9% in 0.3 and 0.6 mg/ml Caffeine withdrawal groups, respectively, p < 0.001) (Fig. 4C), to the level similar to mice who have never been exposed to caffeine (80.6 ± 3.1% as shown in Fig. 1C Before acupuncture in No caffeine group, p > 0.1). Full restoration of the analgesic effect of acupuncture was observed in both groups after caffeine withdrawal (−57.1 ± 6.1% and −46.2 ± 6.0% change in 0.3 and 0.6 mg/ml Caffeine withdrawal groups, respectively, p < 0.001 compared to Before acupuncture), whereas these same mice exhibited slight increases in hypersensitivity by acupuncture in the caffeine conditions on the previous day (+14.3 ± 5.7% and + 27.5 ± 8.8% change in 0.3 and 0.6 mg/ml With caffeine groups, p = 0.05 and p = 0.02 compared to Before acupuncture, respectively) (Fig. 4C,D). As with the previous experiments, sham acupuncture treatment after caffeine withdrawal did not alter the mechanosensitivity (2.4 ± 7.7% and −2.1 ± 9.1% change in 0.3 and 0.6 mg/ml Caffeine withdrawal groups, p = 0.77 and p = 0.83 compared to Before sham acupuncture, respectively) (Fig. 4D). Taken together, the presence of caffeine itself was responsible for inhibiting acupuncture analgesia even at low concentrations, and any enduring effect after the clearance of caffeine from the body does not affect the anti-nociceptive mechanism of acupuncture.

## Discussion

In the present study, we adapted both acute ankle joint and chronic knee joint inflammatory pain models and successfully demonstrated that manual acupuncture treatment induced acute analgesia. Unlike various clinical studies where placebo effects play a major role in overall efficacy of acupuncture treatment, the animals in our experiements had no prior expectations toward the possible outcomes of the procedures. Moreover, our animal models are free from complications in interpreting the effect of caffeine on anlagesia, which can arise from various side effects associated with changes in caffeine consumption in humans. Our data clearly showed that caffeine consumption, ranging from low to high amounts (equivalent to 1–10 cups of coffee a day in humans), interfered with the analgesic action of acupuncture^9, 14^. Systemically administered caffeine acts on adenosine receptors located in various tissues, including in the central nervous and cardiovascular systems, and modulation of these systems changes pain perception^15^. Caffeine also has effects unrelated to adenosine receptors such as inhibition of ryanodine receptors^16^, inositol trisphosphate receptors^17^, phosphodiesterase^18^, and acetylcholinesterase^19^. Caffeine at high doses has been reported to have anti-nociceptive effects in hot-plate and formalin tests, which affect central processing^20^, and was also reported to inhibit acupuncture in postoperative pain^21^. However, our data revealed a new action of caffeine – inhibition of acupuncture-induced analgesia that is mediated by antagonizing peripheral adenosine receptors at the acupuncture point, even at low doses. First, systemic caffeine did not alter the response to mechanical sensation given to the contralateral leg without pain (Fig. 1E), suggesting that caffeine does not alter motor function. Consistently, the dose of caffeine required to affect locomotor activity was much higher (ten times or more) than those used in the present study^14, 22, 23^. Second, a small volume of caffeine administered at the acupuncture point, without raising systemic caffeine concentration, also completely blocked the effect of acupuncture (Fig. 3), indicating that caffeine at the acupuncture point without systemic presence is sufficient to abolish the analgesic effect of acupuncture. Our previous data demonstrated that acupuncture induces analgesia through local activation of the adenosine A1 receptor^1^, and thus caffeine at the site of acupuncture likely blocks receptor activation. Lastly, caffeine withdrawal restored acupuncture’s anti-nociceptive effect (Fig. 4B-D), supporting the notion that the A1 receptor was competitively antagonized by caffeine at the acupuncture site, but was not indirectly inhibited by irreversible systemic changes.

We observed that caffeine’s IC50 was 0.51 µg/ml (2.6 µM) (Fig. 2D), which in a human is less than the predicted caffeine concentration after drinking one cup of coffee^8, 22^. This value is lower than the reported Ki of caffeine (44 µM)^7^, and it is presumed to block less than 15% of A1 receptors^22^. Previously, we estimated that the extracellular adenosine concentration at the acupuncture point in mice peaked around 1.5 µM, much higher than the Ki of the A1 receptor (0.1 µM)^2^, which indicates that the amount of systemic caffeine is not enough to completely block A1 receptor activation. Therefore, it was surprising that such a small amount of caffeine resulted in the complete inhibition of acupuncture analgesia. Perhaps caffeine accumulates in the local tissue where targeted adenosine A1 receptors reside, or a partial blockage of A1 receptors is sufficient to affect the sensory neuronal activity and inhibit the effect of acupuncture. Indeed, a low dose caffeine was reported to induce significant biological consequences such as cerebral glucose utilization^24^. Shen *et al*. reported that Gq protein-coupled rhodopsins control thermal sensory neurons via TRPA1 channel, which enables fine-tuning thermotaxis behaviors to respond to small changes in temperature less than 1 °C^25^. Perhaps the adenosine A1 receptor, also a Gq protein-coupled receptor, might similarly control nociceptive neuronal signaling. Partial blockage of adenosine receptors in peripheral nociceptive neurons may translate to a significant change in nociceptive signals and central pain perception.

In caffeine-drinking groups, we observed a lower degree of hyper-mechanosensitivity compared to the no caffeine group after pain induction (Figs 1B,C and 2B). This observation is consistent with the previously reported anti-nociceptive action of high doses caffeine in rats^20, 26^. It is unlikely that this reduction in the pre-acupuncture mechanosensitivity can be attributed to caffeine suppressing the development of inflammation at the site of the pain, because previous reports found systemic single administration of high doses caffeine can acutely suppress the pain while the development of inflammation was not suppressed^20, 26^. Consistently, in our study the withdrawal of caffeine elevated mechanosensitivity to the levels in the no caffeine group by next day (Fig. 4C Before acupuncture in Caffeine withdrawal groups compared to Fig. 1C Before acupuncture in No caffeine group). Orally administered caffeine distributes throughout the whole body including the acupuncture point. However, peripheral mechanism mediated by orally administered caffeine acting at the local acupuncture point was not involved in the reduction of basal mechanosensitivity, because *in situ* administration of a high dose of caffeine (100 µM), which elevates caffeine concentration at the acupuncture point but not in the central and cardiovascular systems, failed to decrease basal mechanosensitivity (Fig. 3B *insert*, Before versus After caffeine *in situ* administration). It has been well described that moderate to high doses of caffeine influences components of the central and cardiovascular systems, including blood pressure, vascular tone, alertness, sensory responses, and working memory. For example, 0.3 mg/ml caffeine in the drinking water affected cognitive function in mice^9^; ≥10 mg/kg of caffenine altered rodent locomotor activity^14^; and ≥200 mg enhanced attention in humans^13, 27^. Accordingly, in our study a potent reduction in basal mechanosensitivity was limited to the mice that received the drinking water containing the high doses of caffeine (0.3 and 0.6 mg/ml) (Fig. 1B, Before acupuncture), implying the involvement of systemic changes in the central and cardiovascular systems. Interestingly, with the presence of a high-dose of caffeine, acupuncture increased the mechanosensitivity, which nontheless did not exceed the level of hypersensitivity observed before acupuncture in mice that did not recieve caffeine in the drinking water (Figs 1C and 2B, Before acupuncture in No caffeine group versus After acupuncture in 0.3 and 0.6 mg/ml caffeine groups). Sawynok *et al*. reported that the anti-nociceptive action of caffeine is mediated by norepinephrine signaling in the locus coeruleus and spinal cord^20^. In contrast, several studies reported that acupuncture attenuates norepinephrinergic transmission^28, 29^. Thus, acupuncture may counteract the anti-nociceptive action of caffeine, resulting in an apparent increase of mechanosensitivity.

Caffeine is known to induce mild physical dependency after regular consumption^11–13^, and alter gene expression^30, 31^. Therefore, it is possible that caffeine influences gene expression of A1 receptors, such that there may be changes in the sensitivity to A1 receptor-mediated analgesia. We found no significant alterations in *Adora1* expression in both sciatic nerve DRG and skeletal muscle tissue at the acupuncture point (Fig. 4A). The A1 receptor is expressed only in vascular endothelial cells in skeletal muscle^32^, thus these are not likely the targets of the pain suppression by acupuncture. Moreover, tissue injury elicited increases in *Adora2b* and *Adora3*, but not *Adora1*, in skeletal muscle^33^. However, we cannot exclude the possibility that A1 receptors expressed in connective tissue fibroblasts or *Adora1* gene accumulation at peroneal nerve terminals around ST36 embedded inside the muscle tissue are involved in the analgesic mechanism. Nevertheless, we failed to detect a tissue-level change in *Adora1* expression by pain induction or caffeine treatment, indicating that A1 receptors that mediate acupuncture analgesia remained unchanged even after caffeine habituation. This conclusion was supported by the full restoration of acupuncture analgesia following removal of caffeine (Fig. 4C,D), in accordance with a previous observation demonstrating the full restoration of central depressant effects of adenosine agonists 24 hours after caffeine withdrawal^34^. These studies indicate that caffeine habituation at the doses used in the present study does not alter adenosine receptor signaling. Furthermore, the recovery of the analgesic effect showed that the underlying mechanism of acupuncture analgesia was not influenced by any other systemic or local alteration caused by chronic caffeine consumption as well as any adverse effect caused by withdrawal after habituation.

Given that anti-nociceptive effect of acupuncture was (1) suppressed by caffeine at the acupuncture point, (2) counteractive to the nociceptive action of high dose caffeine intake, and (3) fully recovered after caffeine withdrawal, we collectively identified caffeine as a key manipulator of efficacy of acupuncture analgesia. Although the analgesic effect of acupuncture is its most clinically established effect^35, 36^, several clinical studies have reported a lack of benefit of acupuncture compared to placebo^37–39^. Since no clinical studies on the effect of acupuncture have taken a patient’s daily caffeine consumption into consideration, caffeine intake may have been contributing to the contradictory data in the various clinical studies^37, 40–42^. Moreover, severity of caffeine habits can be an important factor when incorporating acupuncture into treatment plans for chronic pain patients. Our data suggest that even a small residual amount of caffeine in the body, hours after a consumption of a cup of coffee, could potentially reduce the efficacy of acupuncture. For some patients, a temporal withdrawal from caffeine may be associated with undesirable side effects. However, patients who stop consuming caffeine regularly may expereince substancially enhanced efficacy of acupuncture therapy. Clinical studies need to confirm if caffeine interferes with acupuncture-induced analgesia and how long patients need to stop taking caffeine to improve acupuncture effects.

## Methods

### Animals, caffeine administration, and experimental models

C57BL/6 male and female mice (Jackson Laboratory) aged 8–12 weeks were used in this study. For oral administration of caffeine, mice were treated with drinking water supplemented with caffeine (Sigma-Aldrich, St. Louis, MO) at concentrations of 0.1–0.6 mg/ml, whereas mice in the no caffeine group received normal drinking water. In some experiments, caffeine (5 µl, 100 µM) or vehicle (5 µl of saline) was administered at the ST36 acupuncture point at a depth of 1.5 mm using a gastight syringe with a 33 G needle instead of oral administration.

Following one-week treatment of the drinking water without or with caffeine, acute inflammatory pain was induced by an intra-articular administration of complete Freund’s adjuvant (CFA, 10 µl, Sigma-Aldrich, St. Louis, MO) into the left hind limb ankle joint using a syringe with 33 G needle^43, 44^. Mice were further treated with either normal or caffeine-containing drinking water for 3–4 days (Figs 1A and 4A,B). Chronic inflammatory pain was induced by an intra-articular administration of monosodium iodoacetate (MIA, 20 mg/ml, 5 µl, Sigma-Aldrich, St. Louis, MO) into the left hind limb knee joint using a syringe with a 33 G needle^45–48^. All mice were returned to standard care for 3 weeks. During the last 3–4 days, the drinking water was changed to either normal or caffeine-containing water (Fig. 2A). No significant weight loss or gain was observed in animals that received caffeine, pain induction, or acupuncture treatment. The animals were housed in a room with a 12 h day/night cycle. All experiments with live animals were performed in accordance with institutional guidelines at the University of Rochester. All procedures were approved by the University Committee on Animal Resources (UCAR-2014-038).

### Pain evaluation and manual acupuncture

Mechanical sensitivity was measured using the Semmes-Weinstein von Frey Aesthesiometer (Stoelting Co, Wood Dale, IL) touch test, as previously described with slight modification^1^. A flowchart of the experimental procedure is shown in Supplemental Materials. Before beginning the measurement session, animals were acclimated to the acrylic chamber (IITC Life Science, Woodland Hills, CA) for 60 min per day for 2 days. Baseline mechanosensitivity was measured twice on different days to validate the stable, low mechanosensitivity in all mice prior to pain induction administered on the day of the second measurement. The average of the two measurements was recorded as the baseline sensitivity value. Pain-induced hyper-mechanosensitivity was evaluated 3 days (CFA) or 3 weeks (MIA) after administration. For some experiments, measurement sessions were repeated on two consecutive days (3 and 4 days after CFA injection). In the experiments testing local caffeine administration (Fig. 3A), mechanical sensitivity was first measured twice, before and after the acupuncture treatment, without caffeine on the first day. On the next day, mechanical sensitivity in the same mice was re-evaluated twice but with administration of caffeine at the acupuncture point 5 min prior to the acupuncture. As a control group, vehicle (5 µl of saline) was administered prior to the acupuncture instead of caffeine. Similarly, caffeine withdrawal experiments (Fig. 4B) were conducted on two consecutive days during which mechanical sensitivity was evaluated twice, before and after acupuncture treatment, on each day with caffeine treatment (first day) and 24 hours after exchange to normal drinking water (second day).

The evaluation was performed by gently applying a thin filament of 0.04 g force to the hind paw of the animals, and the rapid retraction or tapping of the foot was counted as a positive response^1, 49^ (see Supplemental Materials). A total of six or ten trials were given to a paw in one measurement session with an interval of at least 5 min between each trial. Positive responses in the all trials were recorded, and the data are represented as the percent of positive responses. Percent change in mechanosensitivity by acupuncture treatment was obtained by subtracting values of percent responses before acupuncture from values after acupuncture for each animal. The evaluator of mechanosensitivity did not participate in the behavioral experimental design, oral caffeine assignments, local caffeine administrations, and acupuncture procedure, and thus had no prior knowledge of the animal groups. All behavioral measurements were done at the same time of the day during the 12 hour light cycle.

For acupuncture treatment, animals were placed in a restrainer with the hind leg ipsilateral to the inflammation, secured and loosely stretched (see Supplemental Materials). An acupuncture needle (0.20 mm; DBC Spring, Lhasa OMS, Weymouth, MA) was gently inserted to the ST36 Zusanli point at a depth of 1.5 mm, then slowly rotated for 1 min every 3–4 min for a 20 min session while the mice were awake^1^. For sham treatment, the acupuncture needle was inserted the same way, but then left untouched for 20 min without the rotational manipulation. The animals were immediately transferred back to the chamber and acclimated for at least 15 min before post-treatment mechanosensitivity measurements.

### Measurement of caffeine in plasma

Blood samples collected by submandibular bleeding were immediately mixed with 10 mM EDTA, and plasma was isolated by centrifugation at 1,000 *g* for 10 min. Plasma caffeine concentrations were quantified using an enzyme linked immunosorbent assay (ELISA) kit (Neogen, Lansing, MI) according to the manufacturer’s instructions. Standard curves were generated from serial dilutions of caffeine reference solution (1.0 mg/ml caffeine, Cerilliant, Round Rock, TX) in assay buffer containing mouse plasma. ELISA plates were measured at 650 nm, or 450 nm after supplemental addition of 1 N HCl, with a microplate reader (Benchmark Plus, Bio-Rad, Hercules, CA). Dose-response curve of plasma caffeine versus acupuncture-induced changes in mechanosensitivity was calculated with Prism (GraphPad Software, La Jolla, CA) using Hill slope.

### Real-time PCR for adenosine receptor A1 gene expression

Tissues were harvested from five animal groups: (1) Naïve, (2) four days after CFA administration, (3) same as group 2 but with acupuncture treated on the third day, (4) same as group 3 but with 0.3 mg/ml caffeine oral administration, and (5) same as group 3 but with 0.6 mg/ml caffeine oral administration. Groups 4 and 5 were chronically treated with systemic caffeine as described in the “caffeine administration” section above. Ipsilateral L3-L5 dorsal root ganglia (DRG) and tissue surrounding ST36 acupuncture point (roughly 3 × 3 × 3 mm^3^ of tibialis anterior and extensor muscles attached to tibia, without dermis) were dissected from the mice following perfusion fixation with RNAlater solution (Ambion, Foster City, CA) to preserve RNA integrity. The isolated tissues were submerged in RNAlater solution overnight at 4 °C and then, stored at –80 °C. Tissues were homogenized using FastPrep-24 5 G with CoolPrep Adapter (MP Biomedicals, Santa Ana, CA), which separates tibia from acupuncture point tissue samples. Total RNA was extracted with RNeasy Plus Universal Kit (Qiagen, Valencia, CA). Expression of the adenosine A1 receptor gene, *Adora1*, was assessed using TaqMan Gene Expression Assays (Mm01308023_m1), and the expression levels were normalized to multiple reference genes^50^: glyceraldehyde-3-phosphate dehydrogenase (*Gapdh*), acidic ribosomal protein (*Arbp*) and hypoxanthine-guanine phosphoribosyl transferase (*Hprt*) for DRG, and 18 S ribosomal RNA (*Rn18S*), beta-actin (*ActB*), *Gapdh* and *Hprt* for Acupoint, before calculating relative expression.

### Statistical analysis

One-way ANOVA with Tukey’s multiple comparison procedure was used to compare differences between groups. Paired t-test was used to compare before and after acupuncture within each group. The significance level was set at 0.05 for all comparisons. The analyses were implemented with SAS 9.4 (SAS Institute Inc., Cary, NC). The complete statistical data are presented in Supplemental Materials.

## Electronic supplementary material

Supplementary information 
