# Supplementary material for: Presence of caffeine reversibly interferes with efficacy of acupuncture-induced analgesia
Source: Sci Rep. 2017 Jun 13;7:3397. doi: 10.1038/s41598-017-03542-x (PMC5469855; doi:10.1038/s41598-017-03542-x)
Supplement: Supplementary file 1 — Supplementary information [file 41598_2017_3542_MOESM1_ESM.pdf]

Supplemental Materials for Presence of caffeine reversibly interferes with efficacy of acupuncture-induced analgesia

Takumi Fujita, Changyong Feng & Takahiro Takano

Experimental Procedure

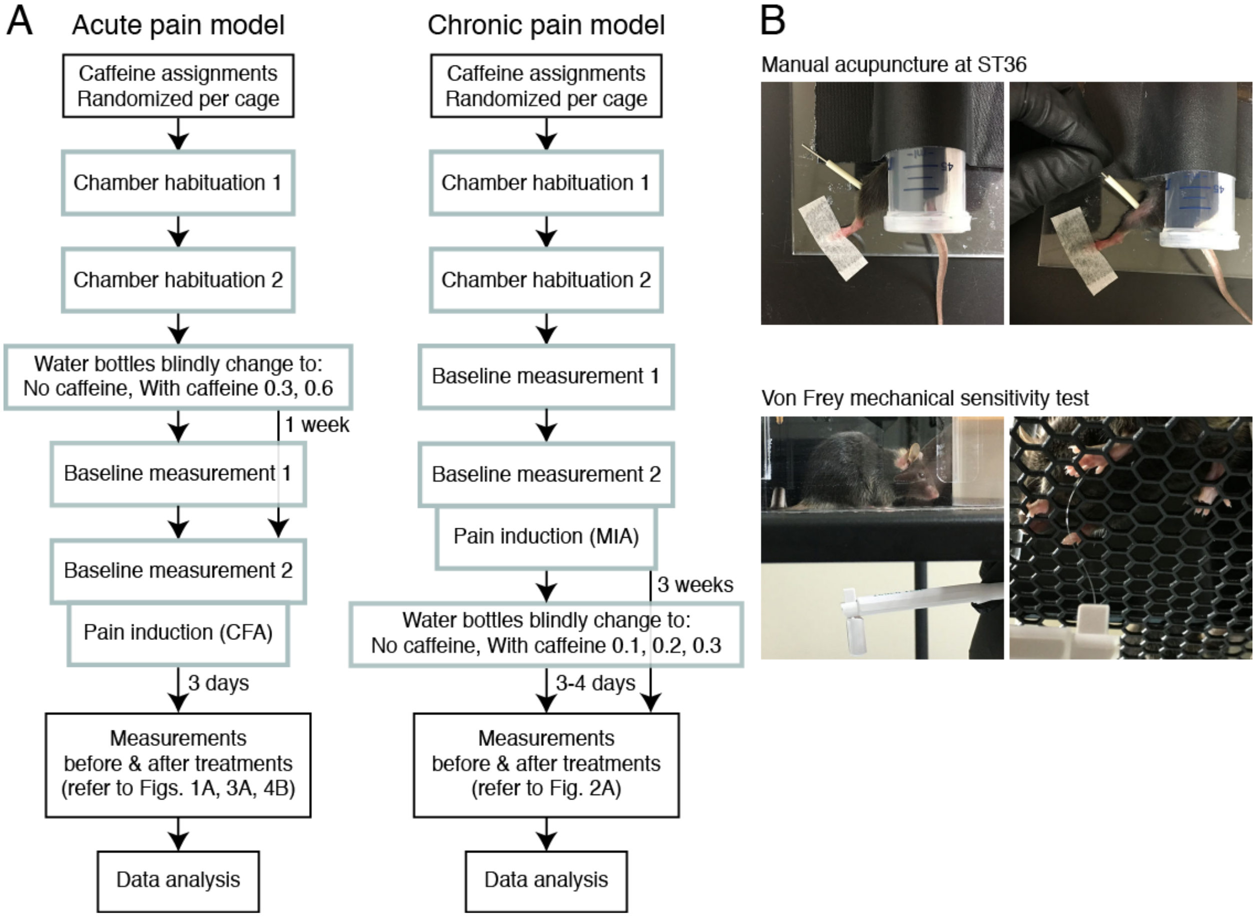

A. A flow chart of the double-blinded randomized experimental procedures. Caffeine doses in drinking water were randomly assigned to each cage containing 4 - 5 mice. Drinking water bottles were prepared such that both the animal caretakers and the evaluator of mechanosensitivity were blind to the caffeine and control groups of animals. Experiemental group assignment was also done before the baseline sensitivity scorings. Regardless of group assignment, all animals received equal pre-scoring chamber habituations twice and baseline measurements twice. Likewise, on the same day after the second baseline measurement, all animals received intra-articular administration of either CFA in the left ankle joint or MIA in the left knee joint. We waited three days for CFA-induced pain to develop, and three weeks for MIA-induced pain, before the acupuncture treatment began. Please refer to Figures 1A, 2A, 3A, and 4B for procedures that are specific to each set of experiments in the part of “Measurements” in this flow chart. Exclusion criteria are: 1) weight loss greater than 10% of original weight, 2) wounds and other external damage, and 3) exceedingly high baseline mechanosensitivity greater than 60%. No animals matched these criteria. Four animals died before the completion of the

experiments.
B. Photographs of manual acupuncture at ST36 and von Frey mechanical sensitivity test.

Statistical data

1. Descriptive statistics of Figure 1B-D:

Variable	Dose = 0			Dose = 0.3			Dose = 0.6		
	N	Mean	Std Dev	N	Mean	Std Dev	N	Mean	Std Dev
Baseline	16	8.1	4.4	16	14.7	9.0	15	12.2	9.7
Before acupuncture	16	80.6	12.4	16	56.9	16.2	15	41.3	19.6
After acupuncture	16	45.6	18.2	16	72.5	21.8	15	59.3	29.6
Change	16	-35.0	14.1	16	15.6	17.9	15	18.0	19.3

Descriptive statistics of Figures 1E:

Variable	Dose =0			Dose =0.3			Dose =0.6		
	N	Mean	Std Dev	N	Mean	Std Dev	N	Mean	Std Dev
Before acupuncture	16	23.1	16.6	16	25.6	18.3	15	18.0	14.7
After acupuncture	16	30.6	20.2	16	35.6	20.0	15	26.0	16.0
Change	16	7.5	21.4	16	10	23.7	15	8	20.1

Descriptive statistics of Figures 1F:

Variable	Dose = 0			Dose = 0.3			Dose = 0.6		
	N	Mean	Std Dev	N	Mean	Std Dev	N	Mean	Std Dev
Before sham	16	75.0	19.2	14	58.3	22.4	14	38.1	17.8
After sham	16	72.9	21.0	14	53.6	22.8	14	33.3	26.1
Change	16	-2.1	18.1	14	-4.8	31.6	14	-4.8	22.1

Descriptive statistics of Figures 1G:

Dose	N	Mean	Std Dev
0	20	0.10	0.21
0.1	19	0.60	0.64
0.2	19	1.28	1.19
0.3	21	2.32	1.77
0.6	16	3.53	2.23

2. Descriptive statistics of Figure 2

Variable	Dose = 0			Dose = 0.1			Dose = 0.2			Dose = 0.3		
	N	Mean	Std Dev	N	Mean	Std Dev	N	Mean	Std Dev	N	Mean	Std Dev
Before acupuncture	14	77.4	15.5	14	76.2	16.9	5	60	14.9	5	60.0	25.3
After acupuncture	14	34.5	12.2	14	73.8	18.2	5	76.7	27.9	5	80.0	18.3
Change	14	-42.9	19.3	14	-2.4	18.3	5	16.7	31.2	5	20.0	13.9

Variable	N	Mean	Std Dev
Before sham	16	75.0	18.3
After sham	16	78.1	18.0
Change	16	3.1	15.2

3.1 Descriptive statistics of Figure 3B, C

Group	Day	Acupuncture	N	Mean	Std Dev
Caffeine *in situ*	3	Before acupuncture	16	80.0	12.1
		After acupuncture	16	43.1	22.1
		Change	16	-36.9	17.8
	4	Before acupuncture	16	78.1	8.3
		After acupuncture	16	73.8	18.2
		Change	16	-4.4	16.3
Vehicle *in situ*	3	Before acupuncture	16	70.0	10.3
		After acupuncture	16	30.0	18.6
		Change	16	-40.0	17.9
	4	Before acupuncture	16	71.9	11.1
		After acupuncture	16	22.5	20.2
		Change	16	-49.4	15.7

3.2 Descriptive statistics of Figure 3B insert

Variable	N	Mean	Std Dev
Before caffeine *in situ*	8	77.1	19.8
After caffeine *in situ*	8	68.8	31.4
Change	8	-8.3	17.8

3.3 Descriptive statistics of Figure 3D

Dose	N	Mean	Std Dev
0	20	0.10	0.21
Caffeine *in situ*	17	0.12	0.18
Caffeine withdrawal (0.3)	15	0.15	0.15
Caffeine withdrawal (0.6)	15	0.18	0.21

4.1 Descriptive statistics of Figure 4A

Variable	N	Mean	Std Dev
No treatment control	8	1	0.15
+CFA	6	0.98	0.23
+CFA +Acupuncture	6	1.26	0.73
+CFA +Acupuncture +Caffeine (0.3)	5	0.96	0.44
+CFA +Acupuncture +Caffeine (0.6)	5	0.65	0.22

4.2 Descriptive statistics of Figure 4C, D

Dose	Acupuncture	Withdrawal status	N	Mean	Std Dev
0.3	Before acupuncture	With caffeine	7	51.4	19.5
	After acupuncture	With caffeine	7	65.7	22.3
	Change	With caffeine	7	14.3	15.1
	Before acupuncture	Caffeine withdrawal	7	84.3	14.0
	After acupuncture	Caffeine withdrawal	7	27.1	11.1
	Change	Caffeine withdrawal	7	-57.1	16.0
	Before sham	Caffeine withdrawal	7	81	17.8
	After sham	Caffeine withdrawal	7	83.3	13.6
	Change	Caffeine withdrawal	7	2.4	20.2
0.6	Before acupuncture	With caffeine	8	31.3	21.7
	After acupuncture	With caffeine	8	58.8	22.3
	Change	With caffeine	8	27.5	24.9
	Before acupuncture	Caffeine withdrawal	8	72.5	16.7
	After acupuncture	Caffeine withdrawal	8	26.3	13.0
	Change	Caffeine withdrawal	8	-46.3	16.9
	Before sham	Caffeine withdrawal	8	77.1	19.8
	After sham	Caffeine withdrawal	8	75	17.8
	Change	Caffeine withdrawal	8	-2.1	25.9
